# SARS-CoV-2: The Impact of Co-Infections with Particular Reference to *Mycoplasma pneumonia*—A Clinical Review

**DOI:** 10.3390/microorganisms10101936

**Published:** 2022-09-29

**Authors:** Silvia Marino, Piero Pavone, Lidia Marino, Giuseppe Nunnari, Manuela Ceccarelli, Claudio Coppola, Chiara Distefano, Raffaele Falsaperla

**Affiliations:** 1 Unit of Pediatrics and Pediatric Emergency, AOU “Rodolico-San Marco”, PO “San Marco”, University of Catania, 95124 Catania, Italy; 2 Section of Pediatrics and Child Neuropsychiatry, Department of Clinical and Experimental Medicine, University of Catania, 95124 Catania, Italy; 3 Neonatal Intensive Care Unit [NICU], AOU “Rodolico-San Marco”, PO “San Marco”, University of Catania, 95124 Catania, Italy; 4 Unit of Infectious Diseases, Department of Clinical and Experimental Medicine, University of Messina, 98122 Messina, Italy; 5 Unit of Infectious Diseases, Department of Clinical and Experimental Medicine, University of Catania, 95124 Catania, Italy; 6 Section of Pediatrics and Child Neuropsychiatry, School of Specialization in Pediatrics, Department of Clinical and Experimental Medicine, University of Catania, 95124 Catania, Italy

**Keywords:** *Mycoplasma pneumoniae*, COVID-19, SARS-CoV-2, respiratory tract co-infection

## Abstract

**Background:** After its 2019 outbreak in Wuhan, scientists worldwide have been studying the epidemiology and clinical characteristics of Severe Acute Respiratory Syndrome Coronavirus 2 (SARS-CoV-2) in children. Evidence indicates that children with SARS-CoV-2 infection are more likely to develop upper and lower respiratory tract infections in association with other infectious agents, such as *Mycoplasma pneumoniae*. Here, we conducted a systematic review of SARS-CoV-2 and *Mycoplasma pneumoniae* co-infection and their clinical course in children. **Methods:** We evaluated the published literature on SARS-CoV-2 by using the medical databases PubMed, Embase, Cochrane Library, Scopus, and Web of Science. In the searches, the Medical Subject Heading (MeSH) terms “SARS-CoV-2 and *Mycoplasma pneumoniae*” AND “co-infection SARS-CoV-2” were used. Studies describing co-infection with SARS-CoV-2 and *Mycoplasma pneumoniae* in children were included in the review. The study was conducted and reported in accordance with the Preferred Reporting Items for Systematic Reviews and Meta-Analyses (PRISMA) guidelines. **Results:** According to the PRISMA guidelines, of the 38 identified studies, 14 were conducted in children (children/adolescents 0–18 years), 6 of which were included in this review. In total, 5867 children under the age of 17 years were diagnosed with SARS-CoV-2 infection through real-time polymerase chain reaction analysis of nasopharyngeal swabs to detect viral RNA. Elevated serum IgM levels specific to *Mycoplasma pneumoniae* were observed in 534 children and were associated with a Kawasaki-like illness in one child. To date, all of the children are alive. **Conclusion:** This study underlines the importance of considering, depending on the clinical context, a possible co-infection between SARS-CoV-2 and atypical bacteria, such as *Mycoplasma pneumoniae.* Co-infections with other respiratory pathogens during the pandemic and hospital stay can cause mistakes in clinical diagnostic and drug treatment. Physicians should perform early differential diagnosis of SARS-CoV-2 in association with other infectious agents. Further studies are needed to have a real incidence of these co-infections and their impact on symptoms, course, and outcome of patients with SARS-CoV-2.

## 1. Introduction

The SARS-CoV-2 virus was responsible for the initial COVID-19 pandemic in 2020 [1]. The disease manifests with respiratory tract and systemic involvement. COVID-19 infection as primary vehicle of the infection was initially assumed to be asymptomatic in children. However, COVID-19 infections, in children are now known to be also potentially paucisymptomatic, presenting with fever, lack of appetite, coughing, and/or involvement of various organs and systems [2,3,4]. The COVID-19 virus is transmitted through direct contact mainly with Flügge’s respiratory droplets. The spread occurs when respiratory droplets released by a patient, when speaking, sneezing, or coughing may subsequently infect the mucous membranes of a susceptible individual. A susceptible individual can also become infected by touching a surface contaminated by the virus and then touching the eyes, nose, or mouth [5]. Real-time reverse transcription-polymerase chain reaction (RT-PCR) of nasopharyngeal swabs is currently the gold standard test for detecting COVID-19 infection. However, in some cases when the RT-PCR test is negative and an underlying COVID-19 infection is suspected, bronchoalveolar lavage may be a useful way to reach a correct diagnosis [6]. Data from the American Academy of Pediatrics report that children represented 18.5% (14,195,580/76,885,307) of all available cases registered [7,8]. In a 2020 systematic survey of patients under 20 years of age affected by COVID-19 infection, 15 to 42% of them resulted asymptomatic [9] and in a report by Forrest et al. [10] on a survey of 82,798 patients under the age of 18 years, 54,948 patients (66%) were asymptomatic, 22,303 (26.9%) were lightly affected, 3781 (4.6%) presented with moderate (moderately severe COVID-19-related clinical signs [including pneumonia, gastroenteritis, dehydration]), and 2% with severe complications requiring intensive care unit (ICU) or mechanical ventilation [10]. In children and adolescents, mortality in cases of COVID-19 directly related to the infection is rare. In Table 1 are reported the pooled data from different Countries obtained for 100.000 children related by COVID-19 deaths. In total, in children/adolescents from 0 to 19 years old affected by COVID-19, the death for this disorder has been valuable at approximately 0.17 × 100.000 affected children [9,11]. In children, the main manifestations of COVID-19 include the involvement of skin, respiratory tract, gastrointestinal tract, and Central Nervous System [9]. Pathogenesis of the COVID-19 infection is related to direct viral infection and to individual immune response. Respiratory tract involvement in cases of COVID-19 infection is thought to occur following three phases: in the first phase, the virus binds to epithelial cells of the respiratory tract and initiates the primary replication, and in this phase the most side of the affected individuals are able to contain the infection and present with mild clinical expression; in the second phase the infection may progress down the airways involving the alveolar epithelial cells and leading to pulmonary viral replication thus causing pulmonary inflammation and pneumonia; in the third phase the rapid replicative progress of the virus at the pulmonary level may cause cellular apoptosis and vascular leakage with release of pro-inflammatory proteins [8,9,10,11,12,13]. In the severe form, the failure of immune response may be linked to insufficient type 1 interferon production or signaling, attenuated or skewed adaptive immune response, which may also be due to viral immune escape properties [14]. Clinical and pulmonary expression differ in patients affected by different variant strains: Yang et al. [15] showed that SARS-CoV-2 Alpha strain mainly affect the lung, while SARS_CoV-2 Omicron affects mainly the upper respiratory tract.

Most respiratory viral pathogens are transmitted through the same route. In children, respiratory viruses are the most common cause of respiratory disorders. The bacterium *Mycoplasma pneumoniae* (*M. pneumoniae*) is the second most common cause of respiratory tract infections. Extrapulmonary involvement has been also related to this bacterium but not clearly established [16]. *M. pneumoniae* species are the smallest known free-living organism. There are over 120 Mycoplasma species: 13 have been isolated from humans but only 4 disorders may present. The disorder may present without any clinical signs, when active the clinical involvement is usually gradual with low-grade fever, malaise, and cough. Upper and lower respiratory tract may be affected. Pneumonia is uncommon, usually self-limited, and its course is usually favorable. The infection may be associated with episodes of skin rush, joint pain, and gastrointestinal symptoms. Hemolytic disorder related to IgM antibodies producing a cold agglutinin reaction has been reported and cardiac involvement with conductive abnormalities on ECG and congestive heart failures may be uncommon but possible complications [15,16,17]. Similar to SARS-CoV-2, *M. pneumoniae* involves mainly the respiratory tract but systemic organs may also be involved [15]. Several cases of co-infection of SARS-CoV-2 with other respiratory agents, including *M.pneumoniae* has been reported in the literature. Here, we conducted a systematic review on the cases of co-infection of SARS-CoV-2 with *M. pneumoniae*, pointing in particular on frequency of this co-infection and on the clinical outcome in children.

## 2. Materials and Methods

**Figure 1:**
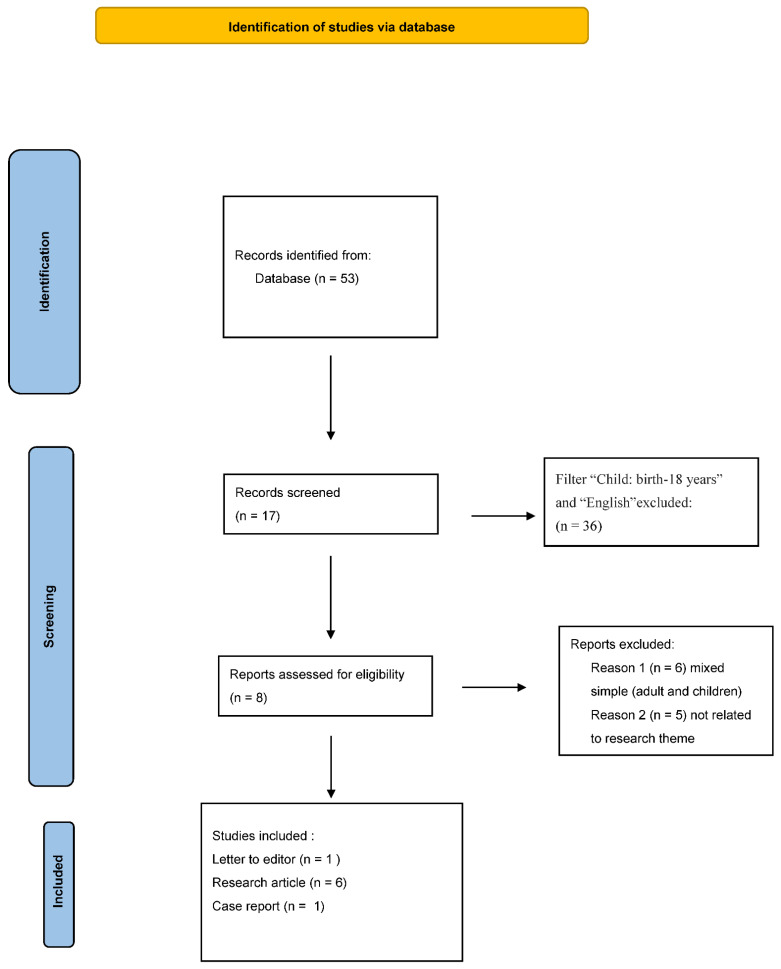
PRISMA Flow-chart.

We evaluated published literature on the topic by using the medical databases PubMed, Embase, Cochrane Library, Scopus, and Web of Science. MeSH terms “SARS-CoV-2 and *M. pneumoniae*” AND “co-infection SARS-CoV-2” were searched. Studies describing co-infection with SARS-CoV-2 and *M. pneumoniae* in children were included. Three reviewers independently screened the titles and abstracts of all citations for eligibility, and retrieved those that met the inclusion criteria. The reference lists of the identified studies were examined to identify reports of interest. Duplicates were identified and removed through independent manual screening performed by two researchers. The study was conducted and reported according to the guidelines of Preferred Reporting Items for Systematic Reviews and Meta-Analyses (PRISMA) flow-chart (Figure 1).

## 3. Results

According to the PRISMA guidelines, of 38 studies, 14 were conducted in children (children/adolescents 0–18 years of age), and 6 were eligible for this review (PRISMA flow-chart). Specifically, three of the studies were retrospective descriptive studies, one was a letter to the editor, one was a research article, and one was a case report. A total of 5867 children under the age of 17 years were tested for SARS-CoV-2 infection with nasopharyngeal swabs and RT-PCR tests. The median age of the children was 6.9 years. No comorbidities were reported. In 534 children, elevated serum IgM levels specific for *M.pneumoniae* were found, and 1 child had an infection associated with a Kawasaki-like disease. To date, all children are alive (Table 2).

## 4. Discussion

### 4.1. The Spectra of Clinical Manifestations and Outcomes of SARS-CoV-2 Infections in Children and Adults Differ

Only a few fatal cases of SARS-CoV-2 in children have been reported in the literature, thus confirming that the mortality rate in children is low [9,10,11]. Reporting data from China maintain that pediatric individuals affected by COVID-19 compared to adults have a better prognosis and a different clinical presentation [26]. Bialek et al. [27] report that among 149,082 (99.6%) laboratory confirmed COVID-19 cases in the USA for which age was known, 2572 (1.7%) presented with an age < 18 years. Among children for which information was available, the authors [27] report that 73% of children had symptoms of fever, cough, or shortness of breath compared with 93% of adults with age between 18 to 64 years during the same period of infection and the number of days in hospital was also lower for children than for adults aged 18 through 64 years. There were three deaths registered among the pediatric cases. In a study conducted by Lu et al. [26] in children under the age of 16 years affected by SARS-CoV-2 and admitted to the Wuhan Children’ s Hospital, the authors report that among the group of 1391 cases tested, a total of 171 (12.3%) were confirmed to be affected by SARS-CoV-2 infection. The median age of the children affected was 6.7 years. In 41.5%, the fever was the main symptom and it was present at any time during the disorder. Other symptoms including cough and pharyngeal erythema were commonly observed. A total of 27 (15.8%) did not show any symptom of infection and no cases of pneumonia were noticed at the radiological investigation. During the hospitalization, three children had care support and invasive care mechanical ventilation all of them presenting with severe systemic disorders including hydronephrosis, leukemia, and intussusception. Lymphopenia was reported in six patients and bilateral ground-glass opacity in 32.7% of the patients. In this study, one death was reported in a 10-month-old child with intussusception and multiorgan failure [26]. Italy was among the countries most affected by SARS-CoV-2, with more than 20,299,013 cases and approximately 170,213 deaths [28]. In children, the infection is reported to be paucisymptomatic, with symptoms similar to infections with other respiratory micro-organisms [28].

### 4.2. SARS-CoV-2 and Co-Infection

Since the beginning of the 2020 pandemic, physicians have focused on diagnosing SARS-CoV-2, while neglecting infections with other respiratory micro-organisms due undoubtedly to the large number of cases under their care. Moreover, the scale of the problem during the early stages of the pandemic keeping people alive was the main priority. However, in some reports differential diagnosis has been discussed.

Wang et al. [29] analyzed 613 patients with fever who underwent multiple test for 13 respiratory pathogens and among these 316 subjects were also tested for 2019-novel coronavirus (2019-nCoV). Among the 316 patients with multiple respiratory pathogens, 104 were positive for 2019-nCov and 6/104 had co-infection with coronavirus (3/104), influenza A virus (2/104), rhinovirus (2/104), and influenza A H3N2 (1/104). The remaining 212 patients the infection involved influenza A virus (11/202), influenza A H3N2 (11/202), rhinovirus (10/202), respiratory syncytial virus (7/202), influenza B virus (6/202), metapneumovirus (4/202), and coronavirus (2/202).

In the report by Ma et al. [30], among 250 hospitalized patients with COVID-19, 39 in addition to this infection showed positive tests for at least 1 respiratory pathogen. A third of these pathogens were detected as early as the first week after the symptom onset, and another third were detected after more than three weeks. The most commonly reported community-acquired pneumonia (CAP) pathogens were Chlamydia pneumoniae (5.2%), respiratory syncytial virus (4.8%), *M. pneumoniae* (4.4%), and adenovirus (2.8%). According to this study [30], patients co-infected with viral pathogens (n = 18) had longer hospital stays when compared to patients co-infected with atypical bacterial pathogens. Except for 1 death, the remaining 38 co-infected patients had a favorable outcome.

A multicenter Italian study was conducted by Garazzino et al. [31] in 168 children, 94 males (55.9%) and 74 (49.15) females, aged 1 day to 17 years all with a confirmed diagnosis of COVID-19 infection. In this group, the mean age was 5 years and among the group 15 were neonates. A total of 33 children (19.6%) showed underlying chronic diseases, including chronic lung disorder (n = 7), congenital malformations or complex genetic syndrome (n = 14), cancer (n = 4) epilepsy (n = 5), gastrointestinal (n = 2), and metabolic disorders (n = 1); seven were immunosuppressed or immunocompromised (n = 7). Except for four children (2.5%) all were symptomatic. The most common symptoms were fever (ranging between 37.5 to 39° C) reported in 82.1% of the cases, cough (48.8%), and also rhinitis (26.8%); gastrointestinal symptoms including vomiting and/or diarrhea was present in 31 children (18.4%) and 5 had seizures. Among the children with seizures, three had a known history of epilepsy, one a past history of febrile seizures and one a first episode of febrile seizure at the onset of COVID-19 infection but not related to SARS-CoV-2 encephalitis and the Cerebrospinal fluid (CSF) analysis was negative. A total of 33 children (19.6%) manifested complications including interstitial pneumonia (n = 26), severe acute respiratory illness (n = 14), and peripheral vasculitis (n = 1). In two patients a preterm neonate and a 2-month-old infant with congenital heart disease it was necessary to admit the child to the intensive care unit (ICU) and treatment with mechanical ventilation. Noninvasive oxygen treatment was carried out on 16 out 168 (9.5%) children and pneumonia was observed either by X-ray or ultrasound in 75 of the children. Co-infection with other pathogens was found in 10 children: 3 with respiratory syncytial virus, 3 with rhino virus, 2 with Epstein-Barr virus, 1 with influenza virus, and 1 with non-SARS coronavirus infection. In a child, a bacterial co-infection with *Streptococcus pneumoniae* was found. According to the authors [31], the hospitalization rate was similar between children with comorbidities and patients without morbidities (23/33 vs. 87/135, respectively: *p* = 0.68, Fisher exact test).

A study was conducted by Li et al. [32] at the aim to assess the impact of SARS-CoV-2 or the prevalence of respiratory pathogens among hospitalized children before and during the COVID-2 epidemic in Shenzhen, China [32]. A total of 5696 children presenting respiratory tract infection were enrolled: 2298 in a period ranged from September to December 2019 and 3398 in the same period of months from September to December of 2020. The patients were distinguished in 4 groups according to the age and year of observation: (a) infant group (1 month–1 year old); 713 cases in 2019 and 1071 in 2020: (b) toddler group (1–3 years old) 641 cases in 2019 and 1036 cases in 2020; (c) preschool group (3–6 years old), 647 cases in 2019 and 932 cases in 2020; (d) school children group (6–14 years old) 297 cases in 2019, and 359 cases in 2020. In 2019, of 2298 specimens, 1850 (80%) were positive for at least one of the 11 pathogens: in 1295 (20%) among the positive patients the occurrence of a single pathogen was found, whereas in 555 (30.0%) of the patients, more than two pathogens were detected. In the following year (2020) in the same period from September to December among 3398 specimens, in 2380 (70%) specimens positivity result was found for at least 1 of the 11 pathogens and in 2034 (85.5%) among the positive specimen a single pathogen was detected and in 346 (14.5%) more than 2 pathogens were detected. The most frequent pathogens found in the period of 2019 were the human rhinovirus, the mycoplasma infection, and the human parainfluenza virus, whereas in the following year human rhinovirus, human respiratory syncytial virus and human parainfluenza virus were prevalent. The influenza detection rate was 5.6% in 2019 and 0% in 2020.

### 4.3. SARS-CoV-2 and M. pneumoniae Co-Infection

During the SARS-CoV-2 epidemic, co- infection related to *M. pneumoniae* has been largely overlooked, despite the disorder being a cause of symptoms similar to those of SARS-CoV-2, including respiratory impairment, skin lesions, non-specific neurological symptoms, and severe neurological complications [33,34,35]. Co-infection SARS-CoV-2 and bacterium *M. pneumoniae* infection has been uncommonly reported during the recent epidemic SARS-CoV-2 infection. From our literature review, we found that of 5.867 infants hospitalized with SARS-CoV-2, 534 had co-infection with *M. pneumoniae* IgM, as shown by elevated *M. pneumoniae* levels.

The bacterium *M. pneumoniae* is a common pathogen causing respiratory diseases in children and the epidemiologic presence of this pathogen among outpatient children with mild respiratory tract infection was studied by Chen et al. [33]. A total of 862 children coming from 15 Centers in China were enrolled. Throat swabs were tested for *M. pneumoniae* RNA, and *M. pneumonia* IgM were tested by a colloidal gold assay. Among the 862 children, *M. pneumoniae* was detected in 78 (90%) children. In this study, decreased infection in COVID-19 prevalence was significantly associated with a reduction of the cases of *M. pneumoniae* infection with a prevalence of r = 0.76, *p* = 0.001.

Co-infection SARS-CoV-2 and *M. pneumoniae* were reported by Serrano et al. [18] in two patients: the first patient, a 48-year-old man had a 5-day history of a widespread urticarial, non-evanescent eruption and fever in association with nonproductive cough and headache. Radiological examination was negative for pneumonia. The second case concerned an 8-year-old boy who had since 7 days from the onset cough and rhinitis and non-palpable purpuric maculopapules on the pretibial region of both the legs.

Gayam et al. [19], in a group of 350 patients with confirmed diagnosis of COVID-19, detected 6 patients who were co-infected with *M. pneumoniae*. The clinical characteristics of these patients consisted of fever present in all the patients, five (83.3%) had a cough, shortness of breath, and fatigue. Other symptoms included myalgia (66.6%), gastrointestinal symptoms (33.3–50%), and altered mental status (16.7%). The chest X-Ray showed at presentation bilateral infiltrates in all of the patients. Electrocardiogram were also reported and one patient died during the hospital course. At the laboratory analysis lymphopenia, elevated erythrocyte sedimentation rate, C-reactive protein, lactate dehydrogenase, interleukin-6, serum ferritin, and D-dimer anomalies were found in all the patients.

Two infants were reported by Li et al. (30] with COVID-19 confirmed infections presenting heart function impaired, and high levels of lactate dehydrogenase and of aspartate-amino-transferase. One of the two infants showed COVID-19 and *M. pneumoniae* co-infection. The authors [20] noticed that the infant with the co-infection COVID-19 and *M. pneumoniae* had a relative longer duration of symptoms, viral duration, and hospitalization compared with the other infants. The authors [20] underline that during the spring season the possibility of co-infection of COVID-19 with other agents as viral respiratory tract infections including *M. pneumoniae* is high and the possibility of co-infection should not be ignored especially in the patients who suffer by severe underlying disorders.

A total of 9 children with a mean age of 8.9 years (range 13 months—14 years) were enrolled in the Pediatric Clinic of Brescia University, Italy with clinical signs of Kawasaki/KSS-like disease [21]. Nasopharyngeal swabs resulted negative in all the children, while in seven out the eight children in which the test was carried out the presence of IgG antibodies against SARSCo-2 was detected. In the group of nine children affected by COVID-19, four showed an increased level of IgM serum against *M. pneumoniae* suggestive of a primary infection. One of these children was tested twice for IgM titer against *M. pneumoniae* and a progressive increase of the IgM level was found during a 7 day period. The four co-infected patients showed a more severe clinical course of the disorder with a more rapid deterioration in term of vasoplegic shock and general clinical conditions. The coexistence of the SARSCoV-2 infection with *M. pneumoniae* may have caused a clinically deleterious effect in the affected patients [21].

One of the first reports of co-infection SARSCoV-2 and *M. pneumoniae* was described by Gao et al. [22] who underlined that co-infection of SARS-CoV-2 and *M. pneumoniae* is uncommon. They reported [22] on a forty-nine-year-old woman who showed symptoms of cough, expectoration, and chest congestion together with elevated C-reactive protein and increased erythrocyte sedimentation rate. CT images displayed ground-glass opacities in bilateral lower lobes and a patchy and striate shadows in the right upper lobe. Diagnosis was confirmed by positivity of the immunoglobulin M antibody of *M. pneumoniae* and positivity for SARS-CoV-2 nuleic acid with real-time fluorescence polymerase chain reaction in the patient’s sputum. Karaaslan et al. (33), among a group of 209 hospitalized children suspected to be affected by SARS-CoV-2, 93 (44.5%) were RT-PCR positive for SARS-CoV-2 infection and 116 (55.5%) were negative at RT-PCR. The children complained mainly of fever (68.8%) and cough (57.0%) and subsequently of headache (10.8%), myalgia (5.4%), sore throat (3.2%), shortness of breath (3.2%), and diarrhea (2.2%), with abdominal pain in one. In seven patients (7.5%) presenting positivity with SARS-CoV-2 co-infections were reported: in two patients COVID-19 with rhinovirus/enterovirus, in two with Coronavirus NL63, in one with adenovirus, and with *M. pneumoniae* in a single patient. Two additional respiratory agents (rhinovirus/enterovirus and adenovirus) were also found in one patient. The authors maintain that co-infected patients are shown to be at a higher risk factor of hospitalization compared with patients exclusively affected by SARS-CoV-2 [23].

In the group reported by Nogueira Lopez et al. [24] COVID-19 diagnosis was confirmed in 33 children by RT-PCR and in 7 by serology tests. Co-infections was found in 7 patients and exactly 5 with *M. pneumoniae*, 1 with parvovirus, and 1 with cytomegalovirus. In 19 (26.4%) of the patients, a worsening of the clinical conditions was noted during the follow up and 14 (19.4%) were in serious conditions and were admitted in the Emergency Department. One patient was initially admitted to hospital but subsequently he had a favorable course. In a retrospective review, Li et al. [25] report on clinical manifestations, laboratory findings, and imaging in a group of patients affected by a single COVID-19 infection (n = 54) and in a group of co-infected pathogens (n = 27). The number of children affected by COVID-19 in association with other pathogens was high (27/81; 33%): the most frequent co-infected pathogen was *M. pneumoniae* (20/81; 25%), and less frequently with virus (6/81, 7%), and bacteria (4/81; 5%). No significant difference between the two groups of patients were found as regards clinical characteristics, laboratory examinations, or hospital stay with lower in white matter counts, neutrophil counts, and lymphocytic counts in the co-infected patients. Chest imaging in co-infected patients showed consolidation in a wider number of cases and the duration of positivity in nucleic acid was shorter. Two points were underlined by these authors [25]: a) co-infection in children with COVID-19 is quite common involving 1/3 of the patients and b) most commonly the co-infection did not cause a significant worsening of the clinical manifestations.

### 4.4. Clinical Influence of COVID-19 and Co-Infection

The role of the co-infection in the clinical course and prognosis of the patients presenting COVID-19 in association with other pathogens requires detailed examination as not many studies looking at the impact of co-infections have been reported. Co-infected children are shown to have a longer durations of symptoms, of viral infection, and of hospitalization than children without co-infections [20,25] but these events have not been constantly reported. A systematic review and meta-analysis was carried up by Scotta et al. [36] who, among 5218 records, screened 43 cases of respiratory viral co-infection. The authors [36] maintain that viral co-infection did not influence risks of the outcome as regards to the length of stay in hospital (mean difference in days in co-infection, −0.10 (95%) confidence interval: −51 to 0.31), length of supplemental oxygen [−0.42 (−1.05 to 0.20)]; need of hospitalization (odds ratio of co-infection 0.96 (95% confidence interval: 0.61–1.51)), supplemental oxygen [0.94 (0.66 to 1.34)], need of intensive care [0.99 (0.64 to 1.54)], mechanical ventilation [0.81 (0.33 to 2.01)], and death [2.22 (0.83 to 5.95)]. The authors conclude [36] that respiratory viral co-infection did not provoke severity in the outcome of the infected patients.

The host immune system plays an important role in the effects of co-infection [37] as during viral co-infection, one virus may interfere with the replication of the other virus, thus resulting in the clearance of one virus and the persistence of the other [38].

In our review, co-infection between SARSCoV2 and *M. pneumoniae* was demonstrated by this study through Real-time PCR and antibody assay. In children, SARS-CoV-2 and Mycoplasma infection may be present but not frequently, and in most of the cases the outcome is favorable without severe complications.

In summary, RT-PCR and antibody assays demonstrated co-infection with SARS-CoV-2 and *M. pneumoniae*, according to this review, and no severe complications were observed.

## 5. Conclusions

According to the results of this study, co-infections with SARS-CoV-2 and atypical bacteria, such as *M. pneumoniae*, should be considered, depending on the clinical context. Clinical diagnostic and therapeutic errors can occur as a result of co-infection with other respiratory pathogens particularly during pandemic hospitalization. According to the literature review, we found that co-infection is associated with longer hospital stays but not with severe outcome and complications. Despite the available data, little information is available regarding the effects of co-infection in patients with SARS-CoV-2. Further studies are needed to determine whether co-infection with SARS-CoV-2 in patients may influence disease outcomes or because of cross-reaction. To determine specific therapies and prevent complications, we believe that screening children with SARS-CoV-2 for other respiratory pathogens is essential and should be sensibly recommended especially in children showing dangerous symptoms or presenting underlying severe disorders. To understand the real incidence of these co-infections and their effects on patients with SARS-CoV-2, more studies are needed.

## Figures and Tables

**Table 1:** Pooled data from different countries for 100.000 children related to COVID-19 deaths [11].

Country	Age Children	Mortality %	Age Children/Adolescents	Mortality%
USA	0–4 y	0–84	5–14 y	0–16
UK	0–9 y	0–09	10–19 y	0–29
Italy	0–9 y	0–16	10–19 y	0–17
Germany	0–9 y	0–12	10–19	0–05
Spain	0–9 y	0–18	10–19 y	0–05
France	0–9 y	0–00	10–19	0–05
South Korea	0–9 y	0–00	10–19	0–00

**Table 2:** SARS-CoV-2 and *Mycoplasma pneumoniae* co-infections.

Authors	Sample	Median Age (Years)	SARS-CoV-2 and *M. pneumoniae*	Main Symptoms	Course	Outcome
Serrano et al., 2020 [18] Italy	2	48–8 y	2/2	Cough, skin eruptions	NR	Favourable
Gayam et al., 2020 [19] USA	350	NR	6/350	Fever, Cough and fatigue, myalgia, diarrhea/vomiting, alterated mental status	Acute cardiac and Acute Kidney injury	1 death
Li et al., 2020 [20] China	2	Infant	½	Impaired heart dysfunction	NR	Favourable
Plebani et al., 2020 [21] Italy	9	9 y	4/9	Kawasaki-like disease	Deterioration of vasoplegic shock and of the general condition	Favourable
Gao et al., 2021 [22] China	1	49 y	1/1	Cough expectoration	Chest congestion	Favourable
Karaaslan et al., 2021 [23] Turkey	209	NR	1/7	Fever, cough	NR	NR
Nogueira López et al., 2021 [24] Spain	72	83, 5 months	5/7	Fever, cough	NR	NR
Li et al., 2021 [25] China	81	NR	20/27	Not specified in co-infection Fever, cough, vomiting/diarrhea	NR	No death
Total	728		40/405			

Legend: NR: Not Reported. *M. pneumoniae*: *Mycoplasma pneumoniae*.

## Data Availability

Not applicable.

## References

[1] Umakanthan S., Sahu P., Ranade A.V., Bukelo M.M., Rao J.S., Lf A.-M., Dahal S., Kumar H., Kv D. (2020). Origin, transmission, diagnosis and management of coronavirus disease 2019 (COVID-19). Postgrad. Med. J..

[2] Pavone P., Marino S., Marino L., Cacciaguerra G., Guarneri C., Nunnari G., Taibi R., Marletta L., Falsaperla R. (2020). Chilblains-like lesions and SARS-CoV-2 in children: An overview in therapeutic approach. Dermatol. Ther..

[3] Pavone P., Ceccarelli M., Marino S., Caruso D., Falsaperla R., Berretta M., Rullo E.V., Nunnari G. (2021). SARS-CoV-2 related paediatric acute-onset neuropsychiatric syndrome. Lancet Child Adolesc. Health.

[4] Marino S., Taibi R., Pavone P., Marino L., Falsaperla R. (2021). Neurotropism of SARS-CoV-2 and others Coronavirus in Children: Mechanisms and Clinical Manifestations. Eurasian J. Med. Oncol..

[5] Williams P.C., Howard-Jones A.R., Hsu P., Palasanthiran P., Gray P.E., McMullan B.J., Britton P.N., Bartlett A.W. (2020). SARS-CoV-2 in children: Spectrum of disease, transmission and immunopathological underpinnings. Pathology.

[6] Marino S., Ruggieri M., Falsaperla R. (2020). Is SARSCoV-2 nasopharyngeal swab still a gold standard in children?. Med. Hypotheses.

[7] (2022). Children and COVID-19: State Data Report. A Joint Report from the American Academy of Pediatrics and the Children’s Hospital Association. Summary of Publicly Reported Data from 49 States, NYC, DC, PR, and G. American Academy of Pediatrics. https://covid.cdc.gov/covid-data-tracker/#new-hospital-admissions.

[8] Hernández J.L.J., Orozco I.F. (2021). COVID-19 in Children: Respiratory Involvement and Some Differences With the Adults. Front. Pediatr..

[9] Deville J.G., Song E., Ouellette C.P. COVID-19: Clinical Manifestations and Diagnosis in Children. Uptodate 2022. www.wolterskluwer.com.

[10] Forrest C.B., Burrows M.E.K., Mejias A., Razzaghi M.H., Christakis D., Jhaveri R., Lee G.M., Pajor N.M., Rao S., Thacker D. (2022). Severity of Acute COVID-19 in Children <18 Years Old March 2020 to December 2021. Pediatrics.

[11] CDC COVID Data Tracker. Demographic Trends of COVID-19 Cases and Deaths in the US Reported to the CDC. www.cdc.gov/covid-data-tracker/index.html#demographics.

[12] Zimmermann P., Curtis N. (2020). Coronavirus Infections in Children Including COVID-19: An Overview of the Epidemiology, Clinical Features, Diagnosis, Treatment and Prevention Options in Children. Pediatr. Infect. Dis. J..

[13] Sharma S., Sharma U., Chaudhary A., Naithani M., Naithani P., Prashar S., Sharma B., Nagar P.K., Bhukya P.L., Bhalerao U. (2021). SARS-CoV-2: Insights from the Immunopathogenesis and Current Clinical Diagnosis and Therapeutic Strategies. J. Immunological. Sci..

[14] Richier Q., Plaçais L. (2022). Infection with SARS-CoV-2: A viral or inflammatory disease ? [Pathogenèse de l’infection par le SARS-CoV-2]. Rev. Prat..

[15] Yang N., Wang C., Huang J., Dong J., Ye J., Fu Y., Huang J., Xu D., Cao G., Qian G. (2022). Clinical and Pulmonary CT Characteristics of Patients Infected With the SARS-CoV-2 Omicron Variant Compared With Those of Patients Infected With the Alpha Viral Strain. Front. Public Health.

[16] Abdulhadi B., Kiel J. (2022). Mycoplasma Pneumonia.

[17] Lanao A.E., Chakraborty R.K., Pearson-Shaver A.L. (2022). Mycoplasma Infections.

[18] Serrano J.M., García-Gil M.F., Monferrer J.C., Manrique B.A., Prieto-Torres L., García M.G., Ochoa C.M., Ara-Martín M. (2020). COVID-19 and Mycoplasma pneumoniae: SARS-CoV-2 false positive or coinfection?. Int. J. Dermatol..

[19] Gayam V., Konala V.M., Naramala S., Garlapati P.R., Merghani M.A., Regmi N., Balla M., Adapa S. (2020). Presenting characteristics, comorbidities, and outcomes of patients coinfected with COVID-19 and *Mycoplasma pneumoniae* in the USA. J. Med. Virol..

[20] Li A., Zhou X., Lu W., Zhou Y., Liu Q. (2020). COVID-19 in two infants in China. Immun. Inflamm. Dis..

[21] Plebani A., Meini A., Cattalini M., Lougaris V., Bugatti A., Caccuri F., Caruso A. (2020). Mycoplasma infection may complicate the clinical course of SARS-Co-V-2 associated Kawasaki-like disease in children. Clin. Immunol..

[22] Zhang B.-Z., Wang Y.-D., Liao Y., Zhang J.-J., Wu Y.-F., Sun X.-L., Sun S.-Y., Guo J.-T. (2020). Endoscopic fenestration in the diagnosis and treatment of delayed anastomotic submucosal abscess: A case report and review of literature. World J. Clin. Cases.

[23] Karaaslan A., Çetin C., Akın Y., Tekol S.D., Söbü E., Demirhan R. (2021). Coinfection in SARS-CoV-2 Infected Children Patients. J. Infect. Dev. Ctries..

[24] López J.N., Lozano C.G., Ruiz C.O., García L.A., Falces-Romero I., Calvo C., Hortelano M.G.-L. (2021). Telemedicine follow-ups for COVID-19: Experience in a tertiary hospital. An. De Pediatría.

[25] Li Y., Wang H., Wang F., Lu X., Du H., Xu J., Han F., Zhang L., Zhang M. (2021). Co-infections of SARS-CoV-2 with multiple common respiratory pathogens in infected children. Medicine.

[26] Lu X., Zhang L., Du H., Zhang J., Li Y.Y., Qu J., Zhang W., Wang Y., Bao S., Li Y. (2020). SARS-CoV-2 Infection in Children. N. Engl. J. Med..

[27] Bialek S., Gierke R., Hughes M., McNamara L.A., Pilishvili T., Skoff T., CDC COVID-19 Response Team (2020). Coronavirus Disease 2019 in Children—United States, February 12–April 2, 2020. Morb. Mortal. Wkly. Rep..

[28] Epicentro, Istituto Superiore di Sanità (ISS) Epidemia. COVID-19. Report Esteso ISS COVID-19: Sorveglianza, Impatto Delle Infezioni ed Efficacia Vaccinale Aggiornamento Nazionale 13/07/2022—Ore 12:00. Rome: ISS; 13 July 2022. Italian. https://www.epicentro.iss.it/coronavirus/bollettino/Bollettino-sorveglianza-integrata-COVID-19_13-luglio-2022.pdf.

[29] Wang M., Wu Q., Xu W., Qiao B., Wang J., Zheng H., Jiang S., Mei J., Wu Z., Deng Y. (2020). Clinical diagnosis of 8274 samples with 2019-novel coronavirus in Wuhan. MedRxiv.

[30] Ma L., Wang W., Le Grange J.M., Wang X., Du S., Li C., Wei J., Zhang J.-N. (2020). Coinfection of SARS-CoV-2 and Other Respiratory Pathogens. Infect. Drug Resist..

[31] Garazzino S., Montagnani C., Donà D., Meini A., Felici E., Vergine G., Bernardi S., Giacchero R., Vecchio A.L., Marchisio P. (2020). Multicentre Italian study of SARS-CoV-2 infection in children and adolescents, preliminary data as at 10 April 2020. Eurosurveillance.

[32] Li L., Wang H., Liu A., Wang R., Zhi T., Zheng Y., Bao Y., Chen Y., Wang W. (2021). Comparison of 11 respiratory pathogens among hospitalized children before and during the COVID-19 epidemic in Shenzhen, China. Virol. J..

[33] Chen J., Zhang J., Lu Z., Chen Y., Huang S., Li H., Lin S., Yu J., Zeng X., Ji C. (2022). Mycoplasma pneumoniae among Chinese Outpatient Children with Mild Respiratory Tract Infections during the Coronavirus Disease 2019 Pandemic. Microbiol. Spectr..

[34] Kammer J., Ziesing S., Davila L.A., Bültmann E., Illsinger S., Das A.M., Haffner D., Hartmann H. (2016). Neurological Manifestations of Mycoplasma pneumoniae Infection in Hospitalized Children and Their Long-Term Follow-Up. Neuropediatrics.

[35] Garone G., Reale A., Vanacore N., Parisi P., Bondone C., Suppiej A., Brisca G., Calistri L., Cordelli D.M., Savasta S. (2019). Acute ataxia in paediatric emergency departments: A multicentre Italian study. Arch. Dis. Child..

[36] Scotta M.C., Chakr V.C.B.G., de Moura A., Becker R.G., de Souza A.P., Jones M.H., Pinto L.A., Sarria E.E., Pitrez P.M., Stein R.T. (2016). Respiratory viral coinfection and disease severity in children: A systematic review and meta-analysis. J. Clin. Virol..

[37] Flores C., Valverde S., Weitz J.S. (2012). Multi-scale structure and geographic drivers of cross-infection within marine bacteria and phages. ISME J..

[38] Kumar N., Barua S., Riyesh T., Chaubey K.K., Rawat K.D., Khandelwal N., Mishra A.K., Sharma N., Chandel S.S., Sharma S. (2016). Complexities in Isolation and Purification of Multiple Viruses from Mixed Viral Infections: Viral Interference, Persistence and Exclusion. PLoS ONE.

